# Special Issue: Fungal Nanotechnology 2

**DOI:** 10.3390/jof9050553

**Published:** 2023-05-11

**Authors:** Kamel A. Abd-Elsalam

**Affiliations:** Plant Pathology Research Institute, Agricultural Research Centre, Giza 12619, Egypt; kamelabdelsalam@gmail.com

**Keywords:** fungus-derived nanoparticles, nanoparticle-based fungicides, fungal bioreactors

## Abstract

Fungal nanotechnology provides techniques useful for molecular and cell biology, medicine, biotechnology, agriculture, veterinary physiology, and reproduction. This technology also has exciting potential applications in pathogen identification and treatment, as well as impressive outcomes in the animal and food systems. Myconanotechnology is a viable option for the synthesis of green nanoparticles because it is simple, affordable, and more environmentally friendly to use fungal resources. Mycosynthesis nanoparticles can be used for various purposes, such as pathogen detection and diagnosis, control, wound healing, drug delivery, cosmetics, food preservation, and textile fabrics, among other applications. They can be applied to a variety of industries, such as agriculture, manufacturing, and medicine. Gaining deeper comprehension of the molecular biology and genetic components underlying the fungal nanobiosynthetic processes is becoming increasingly important. This Special Issue aims to showcase recent advancements in invasive fungal diseases caused by human, animal, plant, and entomopathogenic fungi that are being identified, treated, and treated using antifungal nanotherapy. Utilizing fungus in nanotechnology has several benefits, such as their capacity to create nanoparticles with distinctive characteristics. As an illustration, some fungi can create nanoparticles that are highly stable, biocompatible, and have antibacterial capabilities. Fungal nanoparticles may be used in a variety of industries, including biomedicine, environmental cleanup, and food preservation. Fungal nanotechnology is also a sustainable and environmentally beneficial method. Fungi are an appealing alternative to conventional chemical methods of creating nanoparticles because they are simple to cultivate using affordable substrates and may be cultivated under diverse conditions.

## 1. Introduction

Fungal nanotechnology, also known as myconanotechnology, was coined by Rai M. from India in 2009. This term refers to the production and utilization of nanoparticles within the 1–100 nm size range using fungi. FN is particularly useful in fields such as biomedicine, agriculture, and environmental preservation [1,2]. Metal nanoparticles such as silver, gold, copper, and zinc, as well as other substances such as selenium, titanium dioxide, metal sulfides, and cellulose, have been successfully synthesized using mushrooms, Fusarium, Trichoderma, endophytic fungi, and yeast. FN studies explore various synthesis methods, environmental preservation techniques, and prospects. Investigating the process of nanoparticle production and the effect of different factors on metal ion reduction can lead to the development of cost-effective synthesis and nanoparticle extraction methods. Additionally, FN addresses risk assessment, protection, and control of mycogenic nanoparticles. Fungi can produce extracellular enzymes that hydrolyze complex macromolecules and generate hydrolytes. This metabolic ability makes fungi a strong candidate for producing various types of metallic nanoparticles through bioprocessing [3,4].

In this Special Issue, a critical and detailed analysis of the current progress on the application of metal-based nanoparticles for controlling phytopathogenic fungi in agriculture is presented. The following conclusions and future directions are proposed. The progress achieved in the use of metal nanoparticles for the control of phytopathogenic fungi is outstanding, since the studies developed thus far clearly illustrate that these nanoparticles are an excellent alternative to chemical fungicides when it comes to controlling phytopathogenic fungi in agriculture. Among the metallic nanoparticles, Ag nanoparticles have been the most studied as antifungal agents, followed by Cu nanoparticles. These nanoparticles have shown promising activity against different species of plant pathogenic fungi [5]. Additionally, various metal nanoparticles are mycosynthesised for use in agri-food applications from Trichoderma species. Mycogenic nanoparticles, which are generated from fungal cells or cell extracts, can be used as nanofertilizers, nanofungicides, plant growth regulators, and nanocoatings, among other applications. Additionally, Trichoderma-mediated NPs have been used in environmental remediation procedures such as pollutant detection and removal, including that of toxins containing heavy metals [6]. For instance, unique fungicidal activity in an in vitro experiment completely inhibited the growth of the studied plant pathogenic fungi, and under greenhouse circumstances, the symptoms of cotton seedling illness were significantly diminished. Creating a trichogenic ZnONPs form considerably improved its antifungal action. The use of biocontrol agents, such as T. harzianum, may also be a secure method for producing ZnONPs on a medium scale and applying them to the management of fungal diseases in cotton [7]. *P. indica* produced AgNPs quickly, sustainably, and in an environmentally benign manner for the treatment of mucormycosis and antioxidant activity. Results showed that *Pseudomounas indica* metabolites were used to biosynthesize AgNPs, and several cutting-edge techniques were used to describe the biosynthesized AgNPs. Additionally, *R. microsporus, M. racemosus,* and *S. racemosum* showed no resistance to the remarkable antifungal efficacy of the biosynthesized AgNPs [8]. Chi/Ag-NPs with many biological functions have been created. Gram-positive and Gram-negative bacteria were both extremely susceptible to Chi/Ag-NPs’ antibacterial activity. Along with antioxidant activity, they also reported antibiofilm activity. Furthermore, both unicellular and multicellular fungi were susceptible to Chi/Ag-NPs’ promising antifungal action. Due to their biocompatibility and efficient absorption of wound exudates, Chi/Ag-NPs were seen to dramatically accelerate wound healing at non-toxic doses [9]. By optimizing AgNPs concentration on the interaction with *Piriformospora indica*, for both the broth and the agar, it was possible to study chemically produced AgNPs and their impact on fungal symbiont and black rice. This led to an increase in biomass and a larger fungal colony diameter. An organism known as a “Nano-Embedded Fungus” reacted with the optimal AgNPs concentration (300 ppm). Black rice’s growth and productivity were enhanced by *P. indica* inoculation and AgNPs treatment [10].

In conclusion, Fungal Nanotechnology 2 provides an updated and thorough understanding of the green and sustainable production of metal and organic-based nanostructures by various fungal species, as well as an investigation of intracellular and extracellular mechanisms, with a particular focus on the applications of fungal nanotechnology in the biomedical, environmental, and agri-food sectors. Since FN is still in its infancy, major research should be conducted in this field; plants, animals, and people will all benefit significantly from this, and effective and environmentally acceptable methods should be developed.
